# The magnitude and correlates of Parvovirus B19 infection among pregnant women attending antenatal clinics in Mwanza, Tanzania

**DOI:** 10.1186/s12884-017-1364-y

**Published:** 2017-06-07

**Authors:** Mariam M. Mirambo, Fatma Maliki, Mtebe Majigo, Martha F. Mushi, Nyambura Moremi, Jeremiah Seni, Dismas Matovelo, Stephen E. Mshana

**Affiliations:** 1 Department of Microbiology and Immunology, Weill Bugando School of Medicine, P.O.Box 1464, Mwanza, Tanzania; 2 Department of Microbiology and Immunology, Muhimbili University of Health and Allied Sciences, P.O. Box 65001, Dar es Salaam, Tanzania; 3 Department of Obstetrics and gynecology, Weill Bugando School of Medicine, P.O.Box 1464, Mwanza, Tanzania

**Keywords:** Parvovirus B19, Pregnant women, Tanzania

## Abstract

**Background:**

Human parvovirus B19 (B19) infection has been associated with congenital infection which may result into a number of the adverse pregnancy outcomes. The epidemiology and the magnitude of B19 infections among pregnant women have been poorly studied in developing countries. This study was done to establish preliminary information about the magnitude of B19 among pregnant women attending antenatal clinics in the city of Mwanza, Tanzania.

**Methods:**

A cross-sectional study was conducted between December 2014 and June 2015 among 258 pregnant women attending two antenatal clinics representing rural and urban areas in the city of Mwanza. Socio-demographic data were collected using structured data collection tool. Specific B19 IgM and IgG antibodies were determined using indirect enzyme linked immunosorbent assay kits (DRG Instruments GmbH, Germany). Data were analyzed using STATA version 11 software.

**Results:**

The median age of study participants was 21 IQR (19–25) years. Of 253 pregnant women; 116(44.96%), 109(42.25%) and 33(12.79%) were in the first, second and third trimester respectively. The majority 168(66.4%) of women were from urban areas. Of 253 pregnant women, the overall prevalence of IgM was 83(32.8%) while that of IgG was 142(55.0%) among 258 women tested. A total of 50(19.4%) women were positive for both IgG and IgM indicating true IgM positive. History of baby with low birth weight (OR: 10, 95% CI: 1.82–58.05, *P* = 0.01) was independent predictor of B19 IgG seropositivity and being at the third trimester was protective (OR: 0.38, 95% CI: 0.16–0.92, *P* = 0.03). The IgG titers were found to decrease significantly as gestational age increases (Spearman’s rho = −0.2939, *p* = 0.0004)

**Conclusion:**

More than a half of pregnant women in Mwanza city are B19 IgG sero-positive with about one third of these being B19 IgM seropositive. Further studies to determine the impact of B19 infections among pregnant women and their newborns are recommended in developing countries.

## Background

Human Parvovirus B19 also known as B19 is a single stranded DNA virus commonly responsible for hydrops fetalis, intrauterine fetal death, aplastic crisis, spontaneous abortion, acute symmetric polyarthropathy and erythema infectiosum (5th disease) [1–6]. A fetus is more susceptible to B19 infection during the first and second trimester of the pregnancy which coincides with the development of the erythroid precursors [7]. The decrease in the incidence of fetal loss in the third trimester is due to the fetal immune response to the virus [8]. The B19 is commonly transmitted through respiratory secretions, hand to mouth contact, blood transfusion and trans-placental transfer [9].

The magnitude of B19 have been studied in many developed countries [10, 11] whereby the prevalence of specific B19 antibodies among pregnant women has been found to range from 1 to 5% with transmission rate to the fetus of about 17–33% [12]. High transmission rates have been reported to occur during late spring to summer season [13], with other studies reporting the highest peaks during late winter to spring [14]. Previous studies suggest that 50–65% of the women develop natural immunity against B19 [15]. In developing countries; the epidemiology of B19 particularly in pregnant women is not well documented. Seroprevalence of IgM among pregnant women has been found to range between 3.3% in South Africa and 13.2% in Nigeria [16] while that of IgG was found to range from 24.9% in South Africa to 58.4% in Malawi [17].

Tanzania is among African countries where the magnitude of B19 is not known. No report on B19 infection is available in Tanzania therefore; this study for the first time in Tanzania provides the baseline information regarding the magnitude of this infection among pregnant women attending antenatal clinics.

## Methods

The study was conducted in the city of Mwanza between December 2014 and June 2015. Sample size was calculated by Kish Leslie formula using a prevalence of 20% [18] at 95% confidence interval. The study included pregnant women at different gestation ages attending Makongoro (urban) and Karume (rural) antenatal clinics. The distance from urban to rural clinic is about 20 km. All pregnant women whose gestation ages were unknown were excluded from the study. A structured data collection tool was used to collect socio-demographic and obstetric characteristics. Gestational ages were extrapolated from the last normal menstrual period using pregnancy calculator.

### Specimen collection and laboratory procedures

About 4-5mls of blood sample were drawn from each participant and placed in a plain vacutainer tubes (BD, UK). Specimens were taken to the Catholic University and Health allied Sciences/Bugando Medical Centre (CUHAS/BMC) laboratory where sera were extracted and stored at −40 °C until further processing. Specific B19 IgG and IgM antibodies were detected by using commercial indirect enzyme linked immunosorbent assay (ELISA) kits (DRG Instruments GmbH, Germany). All procedures and interpretation were as per manufacturer’s instructions(http://www.drg-diagnostics.de/45-0-DRG+VirologieSerology+ELISAs.html?ItemPage=6).

### Data analysis

The data were entered in the computer using excel software and analysed using STATA version 11(STATA Corp LP, USA). Categorical variables were summarized as proportions while continuous variables were summarized as median with interquartile ranges. Stepwise logistic regression model was done to determine predictors of B19 seropositivity among pregnant women whereby all factors with *P* value of less than 0.2 in univariate analysis were subjected were subjected into multivariate logistic regression analysis. In addition Kruskal-Wallis equality-of-populations rank test was done to compare the median IgG titres in different trimester followed by spearman correlation test to compare the correlation of the gestational age and IgG titres. Predictors with *p*-values of <0.05 at 95% confidence interval were considered statistically significant.

## Results

**Fig. 1:**
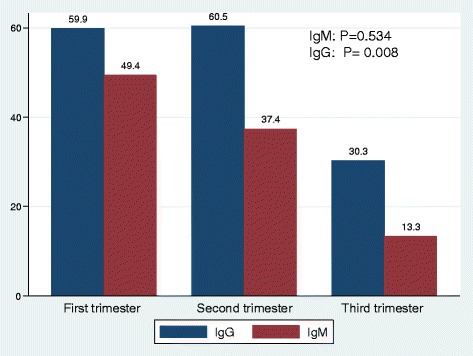
Specific parvovirus B19 IgM and IgG seroprevalence in different trimesters

A total of 258 pregnant women were enrolled into the study. The median age was 21 (IQR: 19–25) years. Out of 258 women 116(45.0%) were in the first trimester, 109(42.3%) in second trimester and 33(12.8%) in third trimester. A total of 168(66.4%) women represented the urban population. Only 253(98%) sera were available for specific parvovirus B19 IgM antibodies testing. The seroprevalence of IgM and IgG B19 antibodies were 83/253(32.8%) and 142/258(56.1%) respectively. Out 253 women, 79(30.6%) were negative for both IgG and IgM B19 antibodies while 50(19.4%) were positive for both IgG and IgM, giving the possibility that the true IgM positive was about 19.4%. Pregnant women in their first and second trimester had significantly higher IgG seropositivity rates than those in the third trimester (Fig. 1).

Out of 85 women from rural areas, 35(41.2%) were B19 IgM seropositive compared to 48(28.6%) of 168 women from urban areas (*P* = 0.045). On multivariate logistic regression analysis none of the factor was found to independently predict B19 IgM seropositivity (Table 1).

**Table 1:** Univariate and multivariate logistic regression analysis of factors associated with IgM seropositivity

Characteristics	IgM seropositivity (*N*, %)	Univariate		Multivariate	
		OR(95% CI)	*P* value	OR(95% CI)	*P* value
Age(years)^a^	21IQR (19–25)	0.97(0.92–1.02)	0.261		
Gestation age					
First trimester (116)	41(35.3)	1			
Second trimester (109)	31(28.9)	0.74(0.42–1.31)	0.310		
Third trimester (33)	11(36.7)	1.05(0.45–2.44)	0.893		
Number in household	3IQR (2–5)	1.04(0.91–1.19)	0.528		
Residence					
Urban (168)	48 (28.6)	1			
Rural (85)	35 (41.2)	1.75(1.01–3.02)	0.045	1.60(0.91–2.8)	0.098
Education					
High (50)	15 (30.0)	1			
Low (203)	68 (33.5)	1.175(0.60–2.30)	0.637		
Occupation					
Employed (178)	59 (33.2)	1			
No employment (75)	24 (32.0)	0.949(0.53–1.68)	0.859		
HIV status					
Negative(95)	31 (32.6)	1			
Positive (8)	4 (50.0)	2.81(0.54–14.66)	0.219		
Unknown (150)	48 (32.0)	1.20(0.72–2.00)	0.483		
History of premature baby					
No (241)	75 (31.12)	1			
Yes (12)	8 (66.67)	4.42(1.29–15.15)	0.018	3.03(0.68–13.42)	0.129
History of miscarriage					
No (221)	71 (32.1)	1			
Yes (32)	12 (37.5)	1.057(0.50–2.22)	0.883	0.91(0.34–2.41)	0.847
History of baby with malformation					
No (247)	80 (32.4)	1			
Yes (6)	3 (50.0)	2.087(0.41–10.57)	0.374		
History of a baby with low birth weight					
No (229)	71 (31.0)	1			
Yes (24)	12 (50.0)	2.22(0.95–5.14)	0.064	1.35(0.46–4.72)	0.098

High: College and above; Low: Primary education

^a^Median

Of 234 pregnant women with no history of low birth weight baby; 121(51.7%) were IgG seropositive compared to 21(87.5%) of those with history of low birth weight baby (OR 6.5, 95% CI; 1.9–22.5), *p* = 0.003. Being at the third trimester (OR 0.38, 95% CI 0.16–0.92, *p* = 0.030) and a history of baby with low birth weight (OR 10, 95% CI 1.82–58.05, *p* = 0.010) were found to be associated with B19 IgG seropositivity on multivariate logistic regression analysis (Table 2).

**Table 2:** Univariate and multivariate regression analysis of factors associated with IgG seropositivity

Characteristics	IgG seropositivity (*N*, %)	Univariate		Multivariate	
		OR(95% CI)	*P* value	OR(95% CI)	*P* value
Age(years)^a^	21IQR(19–26)	1.000(0.956–1.046)	0.984		
Gestation age					
First trimester (116)	66(56.9)				
Second trimester(109)	66(60.5)	1.1(0.68–1.97)	0.578	1.27(0.73–2.2)	0.391
Third trimester(33)	10(30.3)	0.32(0.14–0.75)	0.009	0.38(0.16–0.92)	**0.033**
Number in household	4IQR(2–5)	1.12(0.98–1.281)	0.094	1.08(0.94–1.25)	0.130
Location					
Urban (168)	88 (52.38)	1			
Rural (90)	54 (60.0)	1.36(0.81–2.29)	0.242		
Education					
High (50)	24 (48)	1			
Low (208)	118 (56.7)	1.42(0.76–2.64)	0.266		
Occupation					
Employed (183)	102 (55.7)	1			
No employment (75)	40 (53.3)	0.91(0.53–1.56)	0.724		
HIV status					
Negative (95)	49 (51.6)	1			
Positive (8)	6 (75.0)	2.82(0.54–14.67)	0.219		
Unknown (155)	87 (56.1)	1.20(0.72–2.00)	0.483		
History of premature baby					
No (246)	133 (54.1)	1			
Yes (12)	9 (75.0)	2.54(0.67–9.64)	0.168	0.66(0.08–5.15)	0.697
History of miscarriage					
No (226)	124 (54.9)	1			
Yes (32)	18 (56.3)	1.06(0.51–2.22)	0.883		
History of baby with malformation					
No (252)	138 (54.8)	1			
Yes (6)	4 (66.7)	1.65(0.30–9.18)	0.566		
History of baby with low birth weight					
No (234)	121 (51.7)	1			
Yes (24)	21 (87.5)	6.54(1.90–22.51)	0.003	10(1.82–58.05)	**0.010**

High: College and above; Low: Primary education

^a^Median

### IgG titres by gestational age

**Fig. 2:**
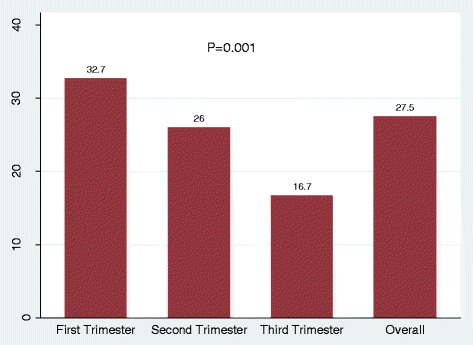
Bar chart showing B19 IgG median titres by trimesters

**Fig. 3:**
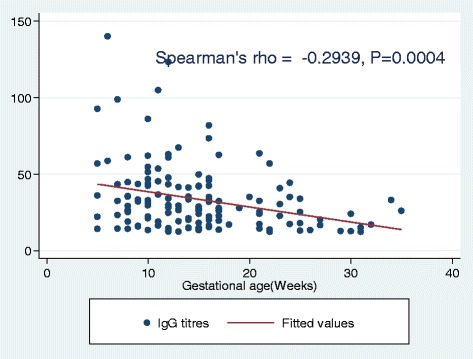
Scatter diagram showing the correlation of B19 IgG titres and gestational age

The median IgG titres among IgG seropositive pregnant women were 27 IU/ml (IQR 17.8–42.2). The median IgG titres by trimesters were 32.7 IU/ml (IQR; 22.1–47.6) for the first trimester, 26 IU/ml (IQR; 17.3–35) for the second trimester and 16.7 IU/ml (IQR; 12.8.1–47.623.9) for the third trimester (Fig. 2). Significantly higher median titres were observed in the first trimester than in the second and third trimesters. Using Kruskal–Wallis equality of population rank test the differences observed were statistically significant (*P* = 0.001). In addition, it was further observed that as gestation age increases the titres were found to decrease significantly (Fig. 3; Spearman’s rho = −0.2939, *p* = 0.0004).

## Discussion

To the best of our knowledge this is the first report on the magnitude of parvovirus B19 infection among pregnant women in Tanzania. The most important findings in this study was that more than half of pregnant women were B19 IgG sero-positive with more than a third of them being B19 IgM sero-positive indicating recent infections. The overall prevalence of both IgM and IgG antibodies which signifies true B19 recent infections was found to be slightly higher compared to many other studies [16–20]. A slightly higher rate in this study could be due to variations in the geographical location, weather and season. The current study was conducted between December and June which is predominantly a rainy season associated with cold weather; all these factors have been found to influence transmission of B19 virus [13].

The high B19 IgM seropositivity found in this population could be due to false positive IgM results. In the current study, B19 IgM positive samples were not confirmed by IgM μ-capture ELISA or by specific B19 polymerase chain reaction (PCR) assay due to limited resources. Samples which were IgM positive and IgG negative, may suggest false-positive IgM reactivity [21]. Therefore, the true IgM positive results in this study could be 19.4% which is slightly higher compared to previous studies as detailed above. The other limitation was failure to collect additional information like history of fever and rashes which could have justified the high prevalence in case of possible outbreaks.

The B19 IgM sero-positivity observed in our study indicates the recent infections in this vulnerable population with the possibility of the adverse pregnancy outcome. This could further be explained by the fact that, in the current study women with history of a baby with low birth weight were more likely to be B19 IgG sero-positive. Further studies to investigate the role of maternal B19 infections in abortion, intra-uterine fetal death and other complications including premature labor and low birth weight are warranted in developing countries.

The majority of women in the present study were IgG sero-positive, our results are within 95% confidence interval of the magnitude of natural immunity reported in Libya, Malawi, Tunisia and Kuwait [16, 20, 22, 23]. However; the IgG sero-prevalence obtained in this study was higher than 24.9% which was observed in South Africa [19]. Our data suggest a relatively high transmission rate of B19 which could be explained by geographical variations. It should be noted that 30.6% of pregnant women in the current study were susceptible for acute parvovirus B19 infections hence at risk of the adverse pregnancy outcomes underscoring the routine screening of this virus during pregnancy.

In the present study; as gestational age increases the odds of being B19 IgG seropositive was found to decrease significantly. The decrease in B19 IgG seropositivity with gestation age observed in the current study might be due to pregnancy hemodilution as previously observed [24–26]. The effect of hemodilution was further supported by the observation that IgG titres were found to significantly decrease as gestational increases. However; low sample in women in third trimester could contribute to the significant differences as selection bias. As in the previous study in Brazil [27], there were no association between B19 IgG seropositivity rates and rural or urban location.

## Conclusions

A significant proportion of pregnant women in Mwanza city are B19 IgG seropositive. A history of baby with low birth weight was independent predictor of B19 IgG sero-positivity while being at the third trimester had a protective effect for being IgG seropositive. Further studies should be done to explore the impact of B19 infections in developing countries. This information may influence policy makers to consider the need for routinely B19 screening among pregnant women.

## Abbreviations

CI: Confidence interval
CUHAS/BMC: The Catholic University and Health allied Sciences/Bugando Medical Centre
ELISA: Enzyme linked Immunosorbent assays
IgG: Immunoglobulin G
IgM: Immunoglobulin M
IQR: Interquartile range
IU: International Unit
OR: Odd ratio
PCR: Polymerase chain reaction
